# Combining Magnetization Transfer Ratio MRI and Quantitative Measures of Walking Improves the Identification of Fallers in MS

**DOI:** 10.3390/brainsci10110822

**Published:** 2020-11-06

**Authors:** Nora E. Fritz, Erin M. Edwards, Jennifer Keller, Ani Eloyan, Peter A. Calabresi, Kathleen M. Zackowski

**Affiliations:** 1 Center for Movement Studies, Kennedy Krieger Institute, Baltimore, MD 21205, USA; keller@kennedykrieger.org (J.K.); zackowski4@gmail.com (K.M.Z.); 2 Department of Physical Medicine and Rehabilitation, Johns Hopkins School of Medicine, Baltimore, MD 21224, USA; 3 Program in Physical Therapy and Department of Neurology, Wayne State University, Detroit, MI 48201, USA; 4 Translational Neuroscience Program, Wayne State University, Detroit, MI 48201, USA; eedwards@med.wayne.edu; 5 Department of Biostatistics, Brown University, Providence, RI 02912, USA; ani_eloyan@brown.edu; 6 Department of Neurology, Johns Hopkins School of Medicine, Baltimore, MD 21224, USA; pcalabr1@jhmi.edu

**Keywords:** multiple sclerosis, walking, accidental falls, magnetic resonance imaging

## Abstract

Multiple sclerosis (MS) impacts balance and walking function, resulting in accidental falls. History of falls and clinical assessment are commonly used for fall prediction, yet these measures have limited predictive validity. Falls are multifactorial; consideration of disease-specific pathology may be critical for improving fall prediction in MS. The objective of this study was to examine the predictive value of clinical measures (i.e., walking, strength, sensation) and corticospinal tract (CST) MRI measures, both discretely and combined, to fall status in MS. Twenty-nine individuals with relapsing-remitting MS (mean ± SD age: 48.7 ± 11.5 years; 17 females; Expanded Disability Status Scale (EDSS): 4.0 (range 1–6.5); symptom duration: 11.9 ± 8.7 years; 14 fallers) participated in a 3T brain MRI including diffusion tensor imaging and magnetization transfer ratio (MTR) and clinical tests of walking, strength, sensation and falls history. Clinical measures of walking were significantly associated with CST fractional anisotropy and MTR. A model including CST MTR, walk velocity and vibration sensation explained >31% of the variance in fall status (*R*^2^ = 0.3181) and accurately distinguished 73.8% fallers, which was superior to stand-alone models that included only MRI or clinical measures. This study advances the field by combining clinical and MRI measures to improve fall prediction accuracy in MS.

## 1. Introduction

Multiple sclerosis (MS) is a complex neurogenerative disease targeting the central nervous system. The pathophysiological hallmarks of MS include inflammatory lesions that result in varying degrees of neuronal demyelination, nerve damage and a vast spectrum of subsequent neurological dysfunctions [1]. Consequently, the etiopathology of MS proves challenging and is not fully understood. At present, it remains widely accepted that MS damages the myelin sheath, a protective covering that insulates axons to promote successful nerve signaling [2]. This varying degree of myelin damage can disrupt signal transmission across a large range of systems [3] and manifest as a myriad of pathological symptoms and comorbidities, including walking and balance impairments [4].

Falls are common in the MS population [5] and represent a serious public health issue. Previous work consistently report that 50–60% or more of ambulatory individuals with MS suffer a fall within a two to six-month period [6] and 70% annually [7], which leads to an even greater impairment to the quality of life from resulting physical injuries, activity curtailment [8], institutionalization [9], social isolation [10] and worse reports of mental health [11]. Additionally, the majority of MS fallers will require medical attention after falling [12,13,14], which leads to greater hospitalizations and demand on health-care resources [10]. 

Falls are individual, complex and multifactorial [15] and consequently, identification and prediction of MS fallers who should receive targeted therapy remains challenging. Currently, the most reliable predictor of future falls is a history of falls (i.e., retrospective recall) [15,16], which is highly problematic as it requires the person to fall at least once and does not account for those individuals with low disability who have yet to experience a fall. Stand-alone clinical assessments of walking speed and balance [17,18] are also standard of care for fall-risk screenings. While these individual measures correlate with falls in MS, they face limitations in predictive validity and ability to identify MS fallers before injury from a fall [19,20,21]. This is perhaps due to the multifactorial nature of falls; one measure cannot capture all factors contributing to falls. A wider assessment of clinical performance that includes not only walking, but strength, sensory loss and cognitive function may improve fall prediction. Further, use of imaging information, which can identify early damage to white matter tracts in conjunction with clinical assessment may provide better fall prediction and allow for tailored interventions to individually address fall risk. 

Prior work from our lab has investigated the combination of clinical and demographic data to better understand fall risk in MS. We showed that combining clinical (Timed 25 Foot Walk [T25FW] and Timed Up and Go [TUG]) and demographic measures was more accurate for predicting fall status (i.e., fallers and non-fallers) than clinical factors (i.e., walking measures) alone [22]. However, we did not consider underlying pathology, which may provide disease-specific information and allow for early targeted fall prevention therapies. In studies of underlying pathology from our lab and others, relationships between decreased white matter integrity and poorer motor function were established, but these studies did not consider fall risk. Notably, decreased fractional anisotropy (FA) in the corticospinal tract (CST) [23], cerebellum [24], and corpus callosum (CC) [25] was related to decreased performance on clinical motor measures. Further, our lab has shown that CST magnetization transfer ratio (MTR) is selectively related to quantitative measures of walking in MS [23]. When quantitative measures of strength and walking are combined, they explain more of the variance in brain CST MTR than demographic or Expanded Disability Status Scale (EDSS) measures [23]. A major gap in our knowledge remains; it is unclear if the combination of clinical measures with demographic information and underlying pathology would provide better fall prediction for persons with MS. 

Both clinical assessment of walking and conventional MRI are commonly utilized by neurologists for clinical care in MS to determine disease progression and effectiveness of pharmacologic intervention. To overcome the limitations of prior research and shift the field of neurorehabilitation toward tailored interventions, we examined the utility of tract-specific MRI measures in combination with clinical measures to assess fall risk. Tract-specific MRI, such as diffusion tensor imaging (DTI) and MTR, allows for examination of the myelin microstructure which is impacted by MS and may show structural changes underlying fall risk. Therefore, the objective of this study was to examine the predictive value of clinical measures (i.e., walking, strength, sensation) and tract-specific brain MRI measures, both discretely and combined, to fall status in MS.

## 2. Methods

Thirty-one individuals with relapsing-remitting MS participated in this study. Two individuals had excess movement in their MRI scans and were excluded from analyses. Three individuals performed their Two Minute Walk Test (2MWT) in a different environment than the rest of the participants, so their data for this measure was also excluded. All participants with MS were recruited from the Multiple Sclerosis Center at Johns Hopkins Medical Institution. Participants were included if they had received a clinical diagnosis of relapsing-remitting MS and were ambulatory with or without an assistive device. Participants were excluded if they had experienced a MS relapse within three months of testing, reported corticosteroid use within 30 days prior to testing, or reported a history of peripheral neuropathy or any other orthopedic or neurologic condition that might interfere with strength, sensation and walking testing. All (*n* = 29) participants were able to follow study-related commands. All participants gave written informed consent. The Institutional Review Boards at both Johns Hopkins Medical Institutes and Kennedy Krieger Institute approved the study procedures (Approval #: NA_00068596).

In a single session, demographic information, retrospective falls, disability status (EDSS) and quantitative measures of strength, sensation and walking were collected. Neuroimaging measures were collected within 1 month of demographic and functional strength and walking measures. Such multimodal assessment of impairments that may limit activity and restrict participation is in line with the International Classification of Functioning, Disability, and Health (ICF) for use in neurological rehabilitation assessment [26].

*Demographics.* Participant demographics included age, sex, symptom duration and EDSS (Table 1).

*Falls Assessment.* Falls were measured retrospectively: participants reported a one-month fall history during the single laboratory visit. Participants were categorized as non-fallers (0 falls in past month) or fallers (≥1 fall in past month). 

*Strength Assessment.* Maximal voluntary contraction of bilateral hip flexion, hip extension and hip abduction was assessed with a handheld dynamometer (Hoggan Health Industries, West Jordan, UT, USA). Quantitative strength testing has demonstrated testing reliability and validity in persons with MS [27]. Hip flexion, extension and abduction were collected using the methods described by our laboratory [28]. The average of two trials of each muscle was recorded and summed strength was calculated from the sum of bilateral hip flexion, bilateral hip extension and bilateral hip abduction measurements. Strength examination was limited to hip musculature, as our lab has previously shown that hip weakness contributes to declines in walking speed in persons with MS [29,30], particularly in the weakest individuals [30].

*Sensation Assessment.* In all participants, sensation was quantified bilaterally at the great toe using a Vibratron II device (Physitemp, Huron, NJ, USA). The Vibratron is a reliable, objective, quantitative measure of sensation in individuals with MS [27]. Participants were instructed to identify which of two rods was vibrating using a two-alternative forced choice procedure. The threshold from worse toe was calculated and used for analysis [31]. The Vibratron demonstrates increased sensitivity in detecting declines in sensation that are not otherwise detectable by the EDSS, which is a common clinical rating scale [30].

### 2.1. Walking Measures

*Walk Velocity.* Participants were instructed to walk at their quickest, safe speed across a 20 ft Zeno Walkway (Protokinetics, Havertown, PA, USA), which records footfalls in real-time. Participants completed six walking trials across the mat. Average walk velocity for each individual was calculated using a custom MATLAB program (The MathWorks, Inc., Natick, MA, USA).

*Timed 25 Foot Walk (T25FW).* To examine walking speed, participants were instructed to walk at their quickest, safe speed along a flat 25 ft walkway [32]. Participants completed two walking trials. T25FW final score for each individual was calculated as the average time of the two trials. The T25FW has established reliability [33] and validity [34] and is commonly used in MS clinical trials [35]. The T25FW is a highly recommended measure for assessing walking function in persons with MS [36].

*Timed Up and Go (TUG).* To examine dynamic balance and walking performance, participants were instructed to stand from a chair, walk 10 feet, turn, walk back and return to a sitting position in the chair at their quickest and safest speed without running [37]. The TUG is reliable and clinically relevant in MS [38] as it incorporates functional tasks of turning and transitioning from sitting to standing into walking. 

*Two-Minute Walk Test (2MWT).* To examine walking endurance, participants were instructed to cover as much distance as possible while walking for 2 min. The 2MWT is strongly correlated with the first 2 min of the Six-Minute Walk Test [39], and is a feasible alternative to the Six-Minute Walk Test [39], which has established reproducibility and reliability [40] and is recommended as a core outcome measure for persons with neurologic disorders [41].

*MRI Measures.* We collected all MRI scans on the same 3T Intera scanner (Philips Medical Systems, Best, The Netherlands). A detailed description of our scanning protocol has been published [42,43,44]. Briefly, we collected a 32-direction diffusion-weighted image as well as a MT-weighted image with a MT prepulse applied at 1.5 kHz off resonance to allow for calculation of MTR by the formula ((MToff-MTon)/MToff). 

*Tract Reconstruction.* We used the Fiber Association by Continuous Tracking method [45] in DTI Studio [46] to reconstruct the corticospinal tract bilaterally [42]. After tract reconstruction, we normalized the data using the methods of Reich et al. [43]. Fractional anisotropy (FA) and magnetization transfer ratio (MTR) were calculated with a custom MATLAB program (The MathWorks, Natick, MA, USA). An average of right and left tracts was used for both FA and MTR values.

### 2.2. Statistical Analyses

All analyses were performed in Stata version 11.1 (StataCorp, College Station, TX, USA). Spearman correlations were used to determine the relationship of the corticospinal tract FA and MTR to walking measures, strength and sensation. Forward stepwise multiple logistic regression with Akaike Information Criterion (AIC) was used to determine the set of variables that best fit the data. AIC criterion [47] accounts for the number of predictors used, allowing for comparison of models with multiple variables to assess model goodness-of-fit. A lower AIC value indicates a better fit. Cross-validation error (CVe), calculated using leave-one-out-cross-validation, is reported for each model to indicate how well the model predicts fall status.

## 3. Results

**Figure 1:**
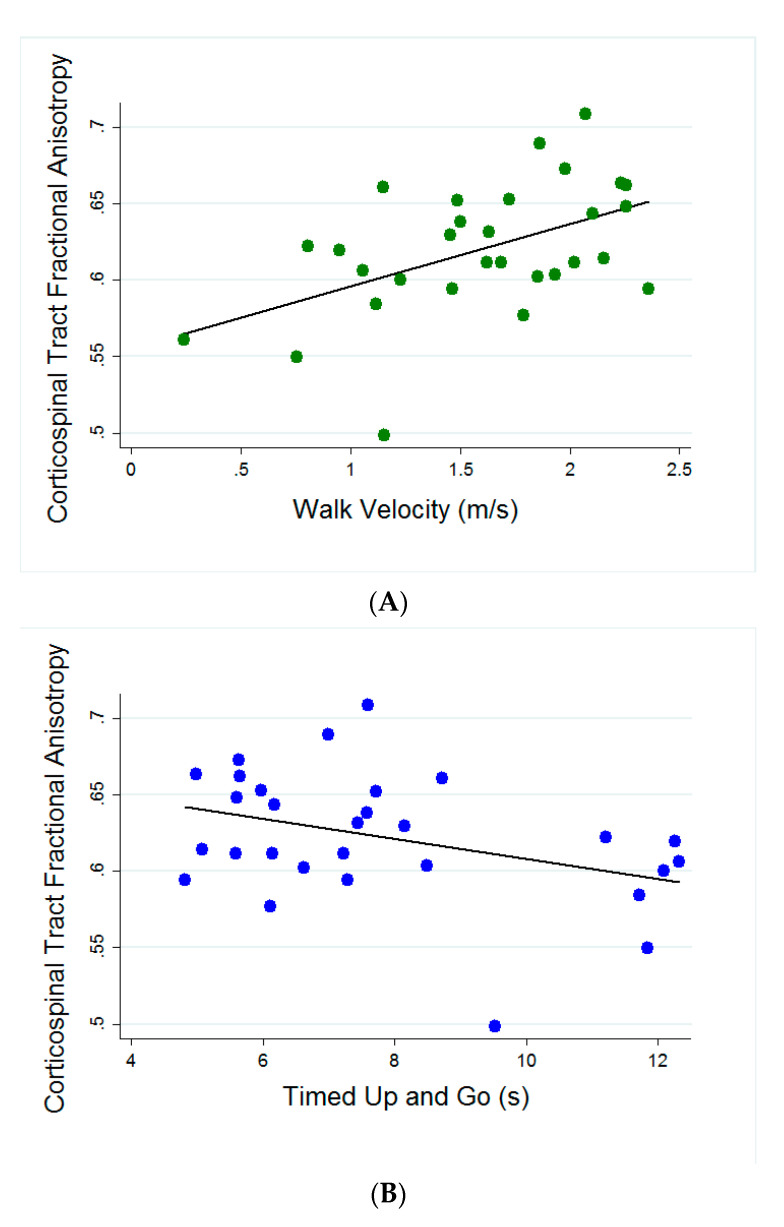

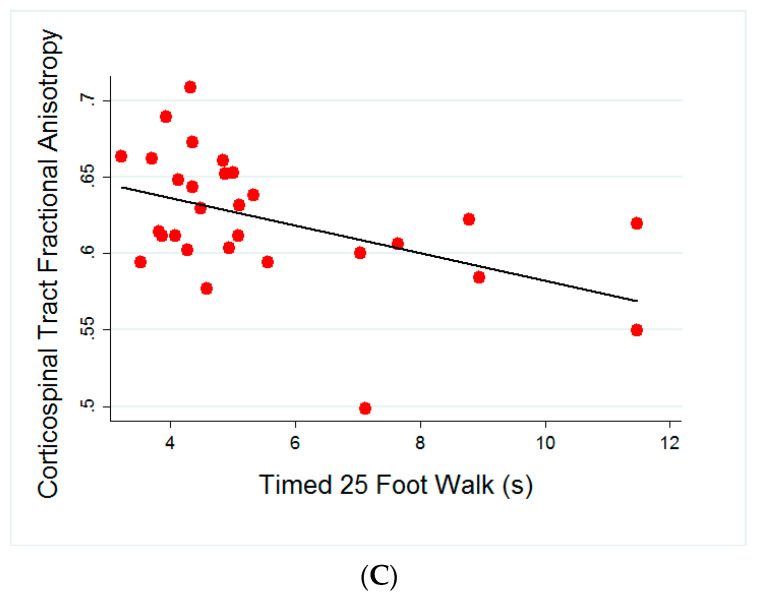
Scatter plots showing significant correlations (*p* < 0.05) of CST FA with: (**A**) Walking velocity (*r* = 0.498), (**B**) Timed Up and Go (*r* = −0.377) and (**C**) Timed 25 Foot Walk (*r* = 0.448).

*Relationships of Corticospinal Tract FA to Quantitative Measures of Strength, Walking, and Sensation.* CST FA was significantly related to walking velocity (*r* = 0.4983; *p* = 0.0051), TUG (*r* = −0.3777; *p* = 0.0434) and T25FW (*r* = −0.4481; *p* = 0.0148) (Figure 1). 2MWT was not significantly related to CST FA (*r* = 0.3372; *p* = 0.0920). There were no significant relationships between CST FA and either summed strength (*r* = 0.170; *p* = 0.386) or vibration sensation (*r* = 0.187; *p* = 0.339). 

*Relationships of Corticospinal Tract MTR to Quantitative Measures of Strength, Walking, and Sensation.* Similar to relationships shown with CST FA shown in Figure 1, CST MTR was significantly related to walking velocity (*r* = 0.3940; *p* = 0.0283) and T25FW (*r* = −0.3831; *p* = 0.0334). Though not significant, TUG (*r* = −0.3383; *p* = 0.0627) and 2MWT (*r* = 0.3789; *p* = 0.0513) were also related to CST MTR. There were no significant relationships between CST MTR and either summed strength (*r* = 0.141; *p* = 0.474) or vibration sensation (*r* = 0.181; *p* = 0.356). 

*Differences between Fallers and Non-fallers.* Although fallers performed more poorly than non-fallers on clinical measures, there were no significant differences between fallers and non-fallers on any MRI or clinical measures (Table 1).

### Predicting Fall Status 

**Figure 2:**
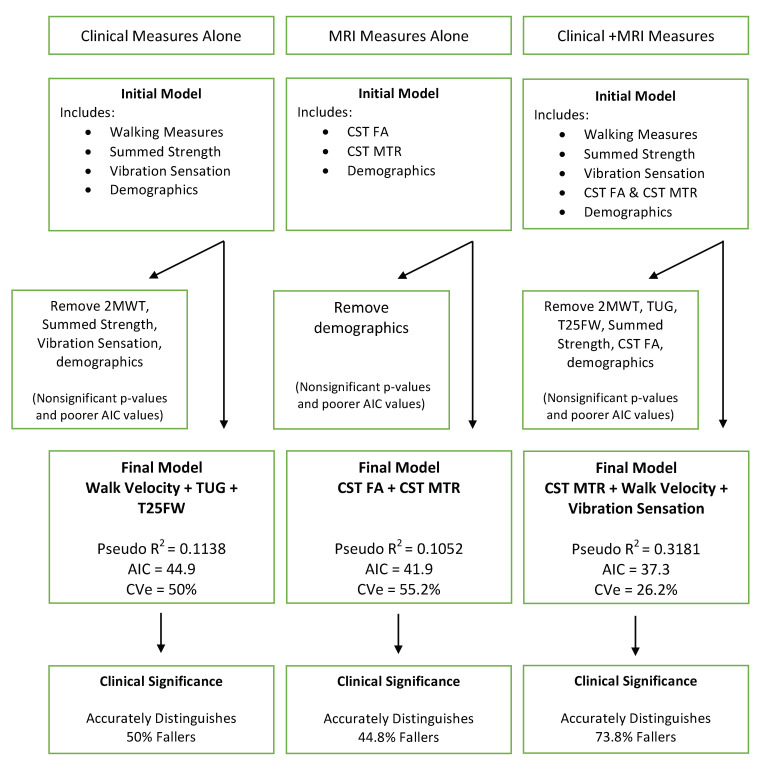
Organizational outline showing the result of three regression models. Akaike information criterion (AIC) were used to determine goodness of fit for each model: Clinical Measures Alone, MRI Measures Alone and the combination of Clinical + MRI Measures on fall status. The model that improved fall classification the most included both MRI and clinical measures, this model explained >26% of the variance in fall status, accurately identifying 73.8% of fallers. Two Minute Walk Test (2MWT); Corticospinal Tract (CST); Cross Validation Error (CVe); Fractional Anisotropy (FA); Magnetization Transfer Ratio (MTR); Timed 25 Foot Walk (T25FW); Timed Up and Go (TUG).

For the model of clinical measures alone, we included all walking measures, summed strength and sensation and demographics. The final model included TUG, T25FW and walk velocity. This model accurately distinguished 50% of fallers (Figure 2). For the model of MRI measures alone, we included CST FA, CST MTR, and demographics; the final model included CST FA and CST MTR and accurately distinguishing 44.8% of fallers. For the model combining both clinical and MRI measures, we included all walking measures, summed strength, sensation, CST MT, CST FA and demographics. The final model included CST MTR, walk velocity and vibration sensation, accurately distinguishing 73.8% of fallers (Figure 2). The combined model represented a better prediction accuracy than models with clinical or MRI data alone.

## 4. Discussion

This study examined the relationship and predictive value of clinical measures of walking, strength, sensation and CST MRI measures to determine whether the combination of clinical and MRI measures would better predict fall status in MS than clinical or MRI measures alone. The critical finding of the current study is that the combination of clinical measures (i.e., quantitative functional assessment) and MRI measures demonstrated 73.8% accuracy (Figure 2), which greatly improves upon the accuracy of stand-alone clinical measures (50%) and stand-alone MRI measures (44.8%) to predict fall status in our sample of MS participants. Additionally, clinical measures of walking performance were significantly associated with MRI measures sensitive to myelin (FA and MTR) in the CST. 

The current stand-alone clinical assessments are plagued by limited predictive accuracy for falls [19,20,21]. These limitations may be attributed to the inability of single measurements to detect varying subtle processes which may underlie the complex event of a fall (i.e., inefficient integration of motor, sensory and cognitive information). Accordingly, fall prevention researchers have highlighted the need for a multifactorial approach [15] for fall risk assessment in MS, which aligns with the multimodal assessment recommended in the ICF Model [26]. Our study sheds light on the importance of combining tests and their respective information to enhance identification of fallers. Our combinatory approach of clinical measures and tract-specific MRI is novel, practical and could markedly improve fall prediction by advancing our understanding of the interactions between disease-specific pathology and clinically observable functions related to falls. 

A significant drawback of the currently used standard of assessing fall risk through fall history is the inability to identify individuals who have yet to experience a fall. Individuals with lower disability may be at risk for falls, but without accurate clinical tools, referrals for rehabilitation to prevent injurious falls may not occur. Our data show that in a cohort of individuals with MS with relatively low disability (EDSS 4.0 on average; Table 1), there were no significant differences between fallers and non-fallers on motor and MRI measures. Yet, when these measures are combined, we see marked improvement in identification of fallers, supporting the idea that multiple measures are needed to understand the multifactorial nature of falls [15]. Another critical finding of the current study was the significant associations between clinical measures of walking performance and MRI measures sensitive to myelin (FA and MTR) in the CST. Specifically, CST FA was significantly related to walking velocity, TUG and T25FW (Figure 1) and CST MTR was significantly related to walking velocity and T25FW. These findings build upon prior studies demonstrating a link between brain pathology and clinically observable function in MS [23,24,25]. Interestingly, there were no significant relationships between CST FA and summed strength. This is in contrast to our prior work that included healthy controls [23] and is likely due to the small, heterogeneous sample of individuals with MS in this study. 

Another novel finding of the current study was the inclusion of sensation in the final combinatory model to predict fall status, which accurately distinguished 73.8% of falls (Figure 2). These data highlight the importance of quantitatively assessing sensory function and studying the unique contributions of sensory dysfunction to fall risk. More than 80% of individuals with MS report sensory problems within 1 year of diagnosis [48] and sensory problems in MS have been linked to a multitude of factors, including walking speed, balance and falls [29,49,50,51]. Despite the significant role sensation plays in clinical impairments such as increased fall risk, clinical tools to accurately measure and monitor sensory problems in MS are limited [52]. Our lab has shown that the Vibratron II is a reliable and objective way to accurately assess sensory function in MS [27]. Further, our recent findings indicate that quantitative vibratory sensation is associated with sensory cortical thickness and thalamic volume in MS [53]. 

### Limitations

Limitations of this study include its small sample size of 29 individuals with relapsing-remitting MS, which may not generalize to ambulatory persons with progressive disease. However, this small-scaled study demonstrates a strong advancement in knowledge regarding fall risk assessment in MS and the potential of combinatory approaches (i.e., clinical and MRI measures, together). This study relied on retrospective recall for falls data collection, which is subject to underestimated reports when compared to prospective reporting [54,55] and is likely attributed to the high prevalence of cognitive dysfunction in MS [56]. Therefore, it is critical that future larger-scale studies leverage the use of technology (i.e., wearable devices, smart phone applications and websites) to accurately report falls. This study was limited to walking, strength and sensation measures and therefore did not assess other factors that could influence MRI measures or fall status, including cognition, daily physical activity, spasticity, and fatigue. Future statistical analyses could include regression models accounting for important co-variates (sex, disease duration, race, physical activity level, spasticity, cognition, etc.) that are also related to functional measures of walking and strength, MRI measures and fall-risk. This study examined one white matter tract of interest, the CST, which is relevant given its critical role in motor control [23,57,58,59]. However, additional regions of interest have been associated with performance on walking and balance measures [24,25]. Our findings did not demonstrate significant relationships among CST MRI measures and summed strength, whereas previous work did [58,59,60]. We attribute this lack of relationship to a small and heterogeneous sample. Lastly, conventional methods of MRI (i.e., DTI and MTR) face limitations in their interpretation of myelination [61,62]. Recent advancements in quantitative neuroimaging techniques have allowed for greater specification of the myelin sheath [63]. Thus, future studies will incorporate additional MRI metrics with increased specific to myelin, such as myelin-water imaging, to advance our understanding of the relationship between myelin damage and fall status in MS. 

## 5. Conclusions

These data show that combining clinical and MRI measures can lead to improved accuracy of fall prediction in people affected by MS. No single measure can address the multifactorial nature of falls, suggesting the importance of larger scale studies to validate the clinical utility of combinatory approaches for assessing fall risk in MS and develop a comprehensive understanding of the relationships among underlying pathology, clinical performance and fall risk. Our future work will incorporate prospective fall reporting to mitigate the inaccuracies of retrospective reporting. We will also use neuroimaging techniques with increased specificity for myelin microstructure (i.e., myelin water imaging). We will explore other white matter regions of interests (i.e., corpus callosum, cerebellum and striatum) and tracts (i.e., dorsal column and medial lemniscus) related to balance and cognitive performance to better characterize neural mechanisms underlying fall risk. Finally, we will assess other factors known to be related to falls in MS, such as cognition, daily physical activity and fatigue. The results of this study, which we regard as a preliminary report, demonstrate that predictive modeling may be a critical element needed to detect subtle abnormalities related to the complex issue of falls in MS. Together, these data provide novel information that can be used to target personalized rehabilitation and ultimately, decrease fall rates for individuals with MS. 

## Figures and Tables

**Table 1:** Study Demographics.

	All MS (*n* = 29)	Fallers (*n* = 14)	Non-Fallers (*n* = 15)	*p*-value
Age (years)	48.7 (11.5)	49.1 (12.1)	48.3 (11.2)	0.841
Sex	17 F; 12 M	8 F; 6 M	9 F; 6 M	0.878
Symptom Duration (years)	11.9 (8.68)	12.3 (9.47)	11.6 (8.20)	0.839
Expanded Disability Status Scale (EDSS)	4.0 [1–6.5]	4.0 [1–6.5]	3.5 [1,2,3,4,5,6]	0.123
Walk Velocity (m/s)	1.64 (0.47)	1.49 (0.44)	1.79 (0.46)	0.0842
Timed Up and Go (s)	7.74 (2.33)	8.35 (2.60)	7.18 (1.97)	0.180
Timed 25 Foot Walk (s)	5.42 (1.99)	6.02 (2.36)	4.86 (1.45)	0.1161
Two Minute Walk Test (m)	161.2 (46.4)	147.2 (44.6)	173.19 (46.0)	0.157
Summed Strength (lbs)	240.1 (84.1)	236.2 (87.1)	243.6 (84.2)	0.817
Vibration Sensation (vu)	5.85 (3.11)	6.06 (3.43)	5.66 (2.89)	0.737
Corticospinal Tract Fractional Anisotropy (CST FA)	0.62 (0.04)	0.62 (0.05)	0.64 (0.03)	0.541
Corticospinal Tract Magnetization Transfer Ratio (CST MTR)	0.46 (0.02)	0.46 (0.02)	0.46 (0.02)	0.275

All values are listed mean (SD), with the exception of EDSS, which is listed median [range]. The *p*-values correspond to testing for a difference in means between fallers and non-fallers performed by using a *t*-test. All *p*-values are corrected for multiple comparisons. Corticospinal Tract (CST); Expanded Disability Status Scale (EDSS); Fractional Anisotropy (FA); Magnetization Transfer Ratio (MTR).
